# Determinants of health-related quality of life decline in interstitial lung disease

**DOI:** 10.1186/s12955-020-01570-2

**Published:** 2020-10-08

**Authors:** Phillen Nozibuyiso Maqhuzu, Boglarka L. Szentes, Michael Kreuter, Thomas Bahmer, Nicolas Kahn, Martin Claussen, Rolf Holle, Larissa Schwarzkopf

**Affiliations:** 1 Helmholtz Zentrum München – German Research Center for Environmental Health (GmbH), Institute of Health Economics and Health Care Management, Comprehensive Pneumology Center Munich (CPC-M), German Center for Lung Research (DZL), Ingolstaedter Landstr. 1, 85764 Neuherberg, Germany; 2 Center for Interstitial and Rare Lung Diseases, Thoraxklinik, University of Heidelberg, German Center for Lung Research (DZL), Röntgenstr. 1, 69126 Heidelberg, Germany; 3 Department of Pneumology and Critical Care Medicine, Thoraxklinik, University of Heidelberg, German Center for Lung Research (DZL), Röntgenstr. 1, 69126 Heidelberg, Germany; 4 LungenClinic Grosshansdorf GmbH Pneumology, German Center for Lung Research (DZL), Wöhrendamm 80, 22927 Großhansdorf, Germany; 5 University Hospital Schleswig-Holstein Campus Kiel, Internal Medicine I, German Center for Lung Research (DZL), Arnold-Heller-Str. 3 /Haus 41a, 24105 Kiel, Germany; 6 Institute for Medical Information Processing, Biometry, and Epidemiology, Marchioninistr. 15, 81377 Munich, Germany; 7 Institut Fuer Therapieforschung (IFT), Leopoldstr. 175, 80804 Munich, Germany

**Keywords:** Diffuse parenchymal lung disease, Patient reported outcomes, K-BILD, EQ-5D VAS, Lung function

## Abstract

**Background:**

Health-related quality of life (HRQL) in interstitial lung disease (ILD) patients is impaired. We aimed to identify baseline predictors for HRQL decline within a 12-month observation period.

**Methods:**

We analyzed 194 ILD patients from two German ILD-centers in the observational HILDA study. We employed the disease-specific King’s Brief Interstitial Lung Disease questionnaire (K-BILD) with the subdomains ‘psychological impact’, ‘chest symptoms’ and ‘breathlessness and activities’, and the generic EQ-5D Visual Analog Scale (VAS). We evaluated how many patients experienced a clinically meaningful decline in HRQL. Subsequently, we investigated medical and sociodemographic factors as potential predictors of HRQL deterioration.

**Results:**

Within the study population (34.0% male, Ø age 61.7) mean HRQL scores hardly changed between baseline and follow up (K-BILD: 52.8 vs. 52.5 | VAS: 60.0 vs. 57.3). On the intra-individual level, 30.4% (n = 59) experienced a clinically relevant deterioration in K-BILD total score and 35.4% (n = 68) in VAS. Lower baseline forced vital capacity (FVC) % predicted determined HRQL decline in K-BILD total score (ß-coefficient: − 0.02, *p* = 0.007), VAS (ß-coefficient: − 0.03, *p* < 0.0001), and in the subdomain ‘psychological impact’ (ß-coefficient: − 0.02, *p* = 0.014). Lower baseline diffusing capacity of carbon monoxide (DLCO) % predicted determined deterioration in ‘breathlessness and activities’ (ß-coefficient: − 0.04, *p* = 0.003) and ‘chest symptoms’ (ß-coefficient: − 0.04, *p* = 0.002). Additionally, increasing age predicted decline in ‘psychological impact’ (ß-coefficient: 0.06, *p* < 0.007).

**Conclusion:**

Around a third of ILD patients experienced a clinically relevant HRQL deterioration in a 12-month period, which was associated with baseline lung function values in all K-BILD domains. As lung function values are time-dependent variables with possible improvements, in contrast to age and ILD subtype, it, thus, seems important to improve lung function and prevent its decline in order to maintain HRQL on the possibly highest level.

## Background

Interstitial lung diseases (ILD) are a group of heterogeneous, rare diseases, which are characterized by pulmonary fibrosis and/or inflammation [1, 2]. ILDs in general are associated with a high burden of disease reflected by a significant loss of years of life and high death rates. The current global burden of disease study estimated an increase in these adverse health outcomes [3]. Treatment consists either of immunosuppressive, anti-inflammatory drugs [4], or anti-fibrotic drugs [5, 6].

The focus of clinical trials has widened from solely prolonging life and delaying disease progression, to also improving the patient’s health status. Health-related quality of life (HRQL) is useful in understanding the impact of treatment and disease on patients’ well-being, functioning and daily life [7]. Well-being is the general state of having good mental health. Functioning encompasses the physical aspects of performing instrumental activities of daily living and daily life refers to the activities and experiences that constitute a person's normal existence [8, 9]. The majority of HRQL studies in ILD focus on idiopathic pulmonary fibrosis (IPF) and sarcoidosis. It is known that HRQL is impaired in IPF [10] and also associated with decline in lung function parameters [11, 12]. However, there is a lack of evidence on HRQL development and comparisons in different ILD entities. In addition, predictors for HRQL decline over time are still sparsely investigated, and studies that investigated HRQL used instruments that are mainly not specific to ILD. Disease-specific questionnaires such as the King’s Brief Interstitial Lung Disease questionnaire (K-BILD), evaluate health status in a wide range of ILDs and assess HRQL in measures that are relevant to ILD. The three domains assessed by K-BILD are psychological impact, chest symptoms, and breathlessness and activities. The two latter domains are the most relevant and capture the burden of ILD in terms of difficulties in breathing and chest discomfort, which are often associated with the symptoms of ILD [13]. For instance, it has been shown that these two domains correlate with lung function [13, 14].

Our study therefore aimed to measure a clinically relevant HRQL deterioration over a 12-months timeframe in different ILD patients using the disease-specific K-BILD, and to identify independent predictors for HRQL decline.

## Methods

### Study design

HILDA (Health Care in ILD Outpatient Visitors) is a prospective, observational study conducted in two large ILD hospitals in Germany, “Thoraxklinik Heidelberg” and “LungenClinic Grosshansdorf” between November 2016 and April 2018. Heidelberg is a city in the South-West of Germany and LungenClinic Grosshansdorf is a small town in the north of Germany. The study successively enrolled 274 patients diagnosed with any ILD subtype who presented to the outpatient practices of the two centers during the first 6 months of the study. Inclusion criteria were patients with a clinically diagnosed ILD, at least 18 years of age, who had given written consent, had a survival forecast of more than 12 months and the patients had to be proficient in the German language in order to fill out the questionnaire. The study collected information from outpatient visits at baseline and after 6 and 12 months. The study collected data on healthcare resource utilization, clinical endpoints, comorbidities and HRQL [14]. For this analysis, we focused on patients with complete HRQL information between baseline and follow up and we therefore included 194 patients in our analyses (70.8% of the initial sample).

### Health-related quality of life instruments

HRQL was assessed by self-administered questionnaires at each visit using the semantically validated German Version [15] of the disease-specific K-BILD and Visual Analog Scale (VAS) of the generic EuroQoL 5 dimensions (EQ-5D-5L). We confined our analyses to the VAS part of the EQ-5D because both VAS and K-BILD instruments use the same scale and can be compared directly. Furthermore, we considered the experience-based VAS (which is assessed in an internationally uniform manner) more suited to reflect subjectively perceived health than the country-specific EQ-5D tariffs. Given previous evidence on good correlation of VAS scores with EQ-5D values in ILD, we assumed the restriction to VAS would not introduce crucial bias [11, 14].

#### K-BILD

K-BILD consists of 15 items and a seven-point Likert response scale, which measure three domains, “psychological impact”, “breathlessness and activities” and “chest symptoms”. Each domain and total score range from zero to 100, with zero being the worst possible health and 100 representing the best health status [13]. The total score is the combined score across all three domains and it based on a pre-defined scoring algorithm that is provided by the authors of K-BILD upon request [13]. The scores are calculated through a predefined algorithm and are not based on a patient-reported scoring algorithm. Additionally, the questionnaire assesses health status during the past 2 weeks. We derived the minimal clinically important differences (MCID) for K-BILD from the revised, longitudinal findings from Patel et al. [16] to determine HRQL decline. This corresponds to sub-domain-specific MCIDs of 7 for “breathlessness and activities”, of 11 for “chest symptoms” and of 6 for “psychological impact” and to MCID of 5 for K-BILD total score [16].

#### EQ-5D-5L and VAS

The EQ-5D-5L consists of five dimensions, mobility, self-care, usual activities, pain/discomfort and anxiety/depression using a five-point Likert scale. VAS is a vertical scale, where the patient reports their present-day health-status on a scale from zero to 100. Likewise, with 100 being the best possible health status [17]. In absence of an ILD-specific MCID, we applied the MCID for Chronic Obstructive Pulmonary Disease (COPD), 6.9, [18] as the two diseases share similar respiratory symptoms [19–21].

### Covariates

We considered possible baseline predictors, such as lung function parameters, diffusing capacity of carbon monoxide (DLCO) % predicted and forced vital capacity (FVC) % predicted, age (in years), sex, disease duration (in years), smoking status (current, former and never-smoker), school education (basic ≤ 9 years, secondary 10–11 years, higher ≥ 12 years), employment status (employed versus unemployed) and ILD subtypes (IPF, sarcoidosis and other ILD subtypes). The ‘other’ ILD subtypes consisted of hypersensitivity pneumonitis (HP), drug-related ILD, combined pulmonary fibrosis and emphysema (CPFE), unclassifiable ILDs and other unspecified forms. A list of all of the full spectrum of ILDs in available in Additional file 1: Table S1. We grouped these subtypes together due to the small individual case numbers. We included baseline HRQL scores to account for the effect of regression to the mean. We determined comorbidity burden as the sum of comorbidities documented by the physician at baseline. Based on previous knowledge on the impact of the number of comorbidities on health outcomes in IPF [22], and also due to the small number of patients diagnosed with each individual comorbid condition, we decided to use comorbidities in one summative index to avoid statistical errors. The pre-specified list included pulmonary hypertension, arterial hypertension, coronary heart disease, congestive heart failure, other cardiovascular disease, diabetes mellitus, emphysema/COPD, lung cancer, depression, gastroesophageal reflux disease, renal failure, obstructive sleep apnea, thromboembolism, and malignant tumors excluding lung cancer). Furthermore, treating physicians had the possibility to include three other comorbidities that were not present in the list. Hence, the highest possible number of comorbidities was 17. We additionally included the use of immunosuppressant medications in the list of possible predictors. Finally, we adjusted for study center location.

### Statistical analyses

We compared patient characteristics of patients who experienced HRQL decline and those who did not using descriptive statistics. As an additional analysis, we investigated the differences in type of medications prescribed at baseline (nintedanib, pirfenidone, immunosuppressant and other medications), disease duration and the sum of comorbidities between the ILD subtypes. We analyzed categorical variables by Pearson’s chi-square test, and continuous variables by the Kruskal Wallis test.

From the 267 eligible patients, 73 (27.3%) had missing HRQL scores at 12 months. Sixty-six patients dropped out and seven patients did not fill out the HRQL questionnaires. We therefore included 194 patients in our analyses.

First, we compared the baseline variables of complete versus incomplete cases to find differences between the two groups. Second, to account for the bias due to drop-out, we performed inverse probability weighting (IPW) on our entire sample. We calculated weights for the 194 complete cases based on the inverse probability of attending the visit after 12 months and filling out the HRQL questionnaire. We modeled the IPW using the selected covariates measured at baseline. Higher weights were assigned to complete cases that were similar to the incomplete cases, and the weighted population therefore resembled the initial cohort at baseline. We then could exclude the incomplete cases from the analyses and perform weighted regression analyses.

We imputed the missing values for FVC% predicted [n = 7 (3.6%)] and DLCO% predicted [n = 20 (10.3%)] by replacing them with the mean values from the sample.

Using MCID, we grouped patients who experienced a clinically meaningful decline in HRQL and those who had not into a binary variable, and we compared the differences between these two groups. Subsequently, we used multivariate logistic regression models to explain the relationship between HRQL decline and the selected covariates for K-BILD total score, K-BILD subdomains, and VAS.

#### Sensitivity analysis

We carried out two sensitivity analyses. First, we analyzed the predictors of HRQL change by performing the regression analyses with a linear outcome for the change in HRQL scores after 12 months. Second, we performed the logistic regressions without weights from IPW to mirror the robustness of our findings in the absence of drop out.

All calculations were performed in a strictly exploratory manner. We did not adjust for multiple testing as HILDA was designed as an epidemiological but not as a confirmatory study with the major aim of hypothesis generation. All statistical analyses were performed using the SAS software package (SAS Institute Inc., Cary, NC, USA, version 9.4) and we considered a *p* value less than 0.05 statistically significant.

## Results

### Comparison of incomplete and complete cases

The incomplete cases had significantly lower FVC% predicted than complete cases (66.8% vs. 74.2%, *p* = 0.009). Furthermore, they had a shorter disease duration than the complete cases (3.0 vs. 4.3 years, *p* = 0.026). There were no other significant differences between these two groups and further information is given in Additional file 2: Table S2.

#### Characteristics of the complete cases

From the complete cases, 66 (34.0%) were male and the mean age was 61.7 ± 12.7 years. Fifty-five (28.4%) patients had IPF, 43 (22.2%) had sarcoidosis and 96 (49.5%) had other ILD subtypes. 114 patients (58.8%) used immunosuppressant medication. The majority of patients were former smokers 112 (57.7%).

Of the 55 IPF patients, 46 (83.6%) were treated with either nintedanib or pirfenidone, and 9 (16.4%) with immunosuppressant medications. Most patients with either sarcoidosis or other types of ILD were treated using immunosuppressive drugs (81.4% and 72.9% respectively). IPF patients had a shorter disease duration than sarcoidosis patients (2.4 ± 1.8 years compared to 8.1 ± 9.8 years). In the heterogeneous group of “other ILDs”, we observed means ranging from 1.7 to 5.3 years, for CPFE and HP respectively, with however large subtype specific variance (min: 0.1 years in CPFE, max: 28.8 years in HP). In addition, IPF patients had a higher mean number of comorbidities than sarcoidosis patients (3.6 compared to 1.9 respectively). These differences were significant and are shown in Additional file 3: Table S3.

### Comparison of HRQL and lung function at baseline and 6 and 12 month follow up

**Fig. 1:**
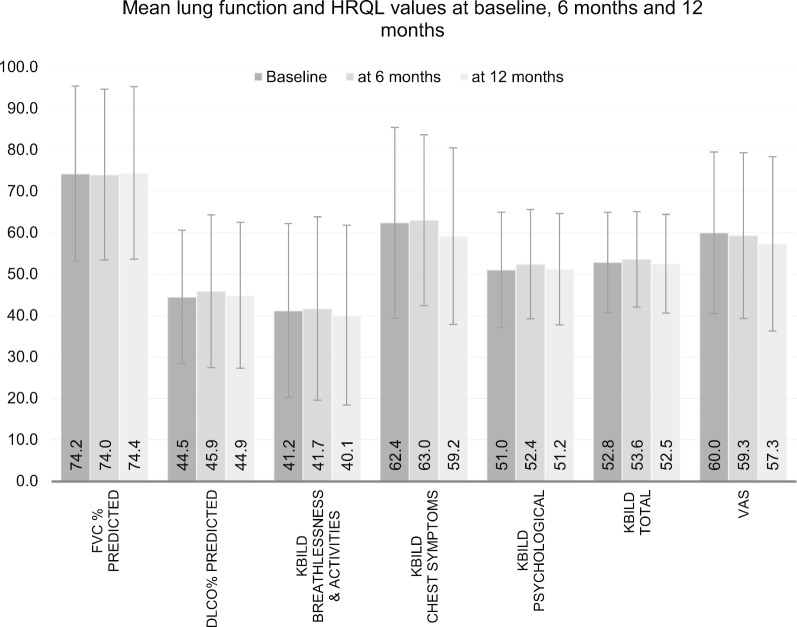
Graph showing differences in mean lung function and K-BILD scores at baseline, 6 months and 12 months of the completed cases

Mean HRQL scores did not change significantly between baseline and at 12-months follow up in the overall cohort (K-BILD total score: 52.8 ± 12.1 vs. 52.5 ± 11.9 | VAS: 60.0 ± 19.5 vs. 57.3 ± 21.0). The K-BILD subdomains remained almost stable, (breathlessness and activities: 41.2 ± 21.0 vs. 40.1 ± 21.7 | chest symptoms: 62.4 ± 23.0 vs. 59.2 ± 21.3 | psychological impact: 51.0 ± 13.9 vs. 51.2 ± 13.4). There was a slight increase in the lung function means after 12 months but these differences were not statistically significant. K-BILD scores were slightly higher in all domains at the 6-month assessment (Fig. 1).

### Decline in HRQL scores and minimal clinically important difference

According to the K-BILD total score, 54.6% (n = 106) of patients experienced HRQL decline. However, considering pre-specified MCIDs, only 30.4% (n = 59) had a clinically relevant deterioration. In comparison, according to VAS 35.4% (n = 68) experienced a clinically relevant HRQL decline. Thirty-two (16.5%) patients deteriorated according to both HRQL questionnaires.

In the subdomains, similarly, 29.4% (n = 57) of patients experienced a clinically relevant deterioration in ‘breathlessness and activities’, 29.9% (n = 58) in ‘chest symptoms’ and 28.4% (n = 55) in ‘psychological impact’. Twenty-one (10.8%) patients deteriorated in all three domains.

### Comparison of patients with HRQL decline to patients without decline or stable HRQL

Patients who deteriorated in K-BILD total score according to MCID presented significantly higher baseline HRQL scores than those who did not deteriorate. The mean baseline K-BILD total scores were 58.2 ± 12.7 versus 50.4 ± 11.1 (*p* = 0.0002) respectively. Mean baseline scores in the K-BILD subdomains were also higher (Table 1). Additionally, participants with worsened K-BILD scores were significantly older, mean age 64.5 ± 12.8 years compared to 60.5 ± 12.4 years (*p* = 0.035). The two groups were however not significantly different according to the mean baseline VAS scores.

**Table 1:** Comparison of patients with HRQL decline and without decline in HRQL

	Worsened HRQL in KBILD total score	Without worsening in HRQL	*p* value
N	59	135	
FVC% predicted, mean (SD)	72.4 (21.7)	75.0 (20.9)	0.594
DLCO% predicted, mean (SD)	41.9 (15.8)	45.7 (16.1)	0.058
Baseline K-BILD breathlessness and activities, mean (SD)	48.7 (21.4)	37.9 (20.0)	*0.001*
K-BILD chest symptoms, mean (SD)	73.5 (23.0)	57.6 (21.3)	< *0.0001*
Baseline K-BILD psychological, mean (SD)	56.4 (15.3)	48.7 (12.6)	*0.002*
Baseline K-BILD total, mean (SD)	58.2 (12.7)	50.4 (11.1)	*0.0002*
VAS, mean (SD)^a^	60.8 (19.0)	59.7 (19.8)	0.743
Mean age, years (SD)	64.5 (12.8)	60.5 (12.4)	*0.035*
Mean time since diagnosis, years (SD)	3.5 (3.8)	4.6 (7.1)	0.778
Mean number of comorbidities (SD)	3.1 (1.6)	2.8 (1.9)	0.119
Female (%)	16 (27.1)	50 (37.0)	0.180
ILD subtypes			0.280
IPF (%)	17 (28.8)	38 (28.2)	
Sarcoidosis (%)	9 (15.3)	34 (25.2)	
Other (%)	33 (55.9)	63 (46.7)	
Smoking status			0.929
Current (%)	2 (3.4)	5 (3.7)	
Former (%)	33 (55.9)	79 (58.5)	
Immunosuppressant medication use (%)	33 (55.9)	84 (62.2)	0.410
Education			0.517
Basic (%)	24 (40.7)	56 (41.5)	
Secondary (%)	20 (33.9)	36 (26.7)	
Higher (%)	15 (25.4)	43 (31.9)	
Employed (%)	16 (27.1)	43 (31.9)	0.510
Center 2 (%)	48 (81.4)	77 (57.0)	*0.001*

^a^The complete cases for VAS were 192 and 70 of them had a clinically meaningful decline in HRQL

The figures in italics are results that are statistically significant with *p* < 0.05

### Predictors of HRQL decline after 12 months

Tables 2 and 3 displays the results of the regression analyses for the determinants of HRQL decline. The predictors for HRQL decline in K-BILD total score were ILD subtype, FVC% predicted, baseline HRQL score and education. Having IPF was linked to less HRQL decline than having other ILDs (ß-coefficient: − 0.979, *p* = 0.032). Lower baseline FVC% predicted was associated with HRQL deterioration (ß-coefficient: − 0.02,* p* = 0.007). Higher baseline HRQL scores were linked to HRQL deterioration (ß-coefficient: 0.095, *p* < 0.0001). In addition, decreasing levels of education predicted HRQL decline, although this result was only significant in patients with a secondary school education compared to those with a higher education (ß-coefficient: 0.949, *p* = 0.027).

**Table 2:** Predictors of HRQL deterioration in K-BILD total score and VAS (weighted)

	K-BILD total			VAS		
	Beta	95% CI	*p* value	Beta	95% CI	*p* value
Baseline FVC% predicted	− *0.022*	*[*− *0.039;*− *0.006]*	*0.007*	− *0.034*	*[*− *0.051;*− *0.017]*	< *0.0001*
Baseline DLCO% predicted	− 0.018	[− 0.042;0.006]	0.131	− 0.006	[− 0.027;0.016]	0.618
Baseline HRQL score	*0.095*	*[0.057;0.134]*	< *0.0001*	*0.038*	*[0.020;0.056]*	< *0.0001*
Age	0.024	[− 0.015;0.062]	0.234	*0.035*	*[0.000;0.070]*	*0.048*
Time since diagnosis	− 0.004	[− 0.062;0.053]	0.880	− 0.019	[− 0.074;0.036]	0.497
Number of comorbidities	0.018	[− 0.189;0.224]	0.868	− 0.014	[− 0.208;0.179]	0.885
Female	0.703	[− 0.011;1.417]	0.054	0.205	[− 0.436;0.846]	0.531
ILD subtype (ref = Other ILD)						
IPF	− *0.979*	*[*− *1.873;*− *0.084]*	*0.032*	− 0.163	[− 0.961;0.635]	0.689
Sarcoidosis	0.169	[− 0.89;1.228]	0.755	0.525	[− 0.461;1.510]	0.297
Smoking (ref = non-smoker)						
Current smoker	− 0.232	[− 2.142;1.678]	0.812	0.706	[− 1.018;2.430]	0.422
Former smoker	− 0.172	[− 0.855;0.512]	0.623	0.100	[− 0.547;0.748]	0.761
Immunosuppressant use	− 0.421	[− 1.224;0.382]	0.304	− 0.274	[− 1.008;0.460]	0.464
Education (ref = higher)						
Basic education	0.302	[− 0.499;1.104]	0.459	− 0.015	[− 0.723;0.694]	0.968
Secondary education	*0.949*	*[0.105;1.792]*	*0.027*	− 0.312	[− 1.086;0.461]	0.428
Unemployed	0.151	[− 0.830;1.133]	0.763	0.498	[− 0.409;1.405]	0.282
Center 2 (ref = center 1)	*1.302*	*[0.482;2.124]*	*0.002*	0.335	[− 0.354;1.024]	0.340

The figures in italics are results that are statistically significant with *p* < 0.05

**Table 3:** Predictors of HRQL deterioration in K-BILD subdomains (weighted)

	K-BILD breathlessness and activities			K-BILD chest			K-BILD psychological		
	Beta	95% CI	*p* value	Beta	95% CI	*p* value	Beta	95% CI	*p* value
Baseline FVC% predicted	0.006	[− 0.010;0.023]	0.444	0.001	[− 0.015;0.018]	0.863	− *0.021*	*[*− *0.037;*− *0.004]*	*0.014*
Baseline DLCO% predicted	− *0.035*	*[*− *0.059;*− *0.011]*	*0.004*	− *0.040*	*[*− *0.065;*− *0.015]*	*0.002*	− 0.010	[− 0.034;0.015]	0.427
Baseline HRQL score	*0.028*	*[0.011;0.045]*	*0.001*	*0.050*	*[0.033;0.068]*	< *0.0001*	*0.084*	*[0.051;0.117]*	< *0.0001*
Age	0.007	[− 0.028;0.042]	0.682	− 0.009	[− 0.049;0.031]	0.665	*0.059*	*[0.016;0.102]*	*0.007*
Time since diagnosis	0.018	[− 0.036;0.073]	0.510	− 0.007	[− 0.067;0.053]	0.821	0.035	[− 0.032;0.101]	0.306
Number of comorbidities	0.113	[− 0.088;0.314]	0.272	0.020	[− 0.193;0.232]	0.856	− 0.091	[− 0.301;0.119]	0.395
Female	0.634	[− 0.037;1.304]	0.064	0.724	[− 0.008;1.456]	0.053	0.332	[− 0.388;1.053]	0.366
ILD subtype (ref = Other ILD)									
IPF	0.272	[− 0.551;1.094]	0.518	− 0.192	[− 1.078;0.695]	0.671	− 1.115	[− 2.018;− 0.213]	0.015
Sarcoidosis	− 0.360	[− 1.363;0.643]	0.482	0.814	[− 0.312;1.939]	0.156	− 0.086	[− 1.208;1.036]	0.880
Smoking (ref = non-smoker)									
Current smoker	− 1.103	[− 3.201;0.995]	0.303	− 0.582	[− 2.581;1.416]	0.568	0.284	[− 1.734;2.303]	0.783
Former smoker	− 0.308	[− 0.947;0.330]	0.344	− 0.522	[− 1.232;0.188]	0.150	− 0.072	[− 0.798;0.654]	0.845
Immunosuppressant use	0.209	[− 0.554;0.971]	0.592	− 0.183	[− 0.985;0.619]	0.654	− 0.533	[− 1.346;0.280]	0.199
Education (ref = higher)									
Basic education	− 0.217	[− 0.945;0.511]	0.559	0.431	[− 0.392;1.253]	0.305	− 0.045	[− 0.834;0.743]	0.910
Secondary education	0.321	[− 0.44;1.082]	0.409	0.811	[− 0.073;1.696]	0.072	0.304	[− 0.556;1.163]	0.489
Unemployed	− 0.443	[− 1.389;0.503]	0.359	0.961	[− 0.117;2.039]	0.080	0.369	[− 0.685;1.423]	0.493
Center 2 (ref = center 1)	0.174	[− 0.553;0.901]	0.639	*1.063*	*[0.252;1.873]*	*0.010*	*0.949*	*[0.125;1.773]*	*0.024*

The figures in italics are results that are statistically significant with *p* < 0.05

Similarly, baseline HRQL scores were significantly associated with HRQL decline in all subdomains and VAS. FVC% predicted determined HRQL decline only in ‘psychological impact’ and VAS. In contrast, lower baseline DLCO% predicted—instead of baseline FVC% predicted—was associated with HRQL deterioration in ‘breathlessness and activities’ (ß-coefficient: − 0.04, *p* = 0.003) and ‘chest symptoms’ (ß-coefficient: − 0.04, *p* = 0.002). In addition, increasing age was associated with HRQL decline in ‘psychological impact’ (ß-coefficient: 0.06, *p* = 0.007) and VAS (ß-coefficient: 0.04, *p* = 0.048).

Our results revealed significant associations between study center and HRQL decline only in the K-BILD questionnaire. VAS, however did not detect any significant associations with study center.

### Sensitivity analyses

In the first sensitivity analysis, the predictors of linear HRQL change and the predictors of HRQL deterioration were similar in terms of lung function and baseline HRQL scores (Additional file 4: Table S4). Higher DLCO% predicted was associated with higher HQRL scores. Secondly, performing the regression analyses without IPW yielded similar results for ‘breathlessness and activities’, and ‘chest symptoms’ and VAS. For ‘psychological impact’, the effect of FVC% predicted, age and center diminished and for K-BILD total score, the effect of FVC% predicted and IPF were not significant (Additional file 5: Table S5).

## Discussion

This study demonstrates HRQL change in a prospective study of patients with ILDs. Our data illustrates that HRQL deteriorated by a clinically relevant amount in a third of the patients after this period. Key predictors for HRQL decline were lower lung function values at baseline across all K-BILD domains. Additionally, older age was a significant determinant in the deterioration of ‘psychological impact’.

In a comparison of the complete and incomplete cases, the incomplete cases (dropouts) had lower lung function values at baseline although they had a shorter disease duration. This could be indicative of progressive fibrosing ILD. Kolb et al. [23] described that, progressive fibrosing ILDs are characterized by a rapid decline in lung function, which may have been the cause for the drop out. Moreover, another study describes the patterns of progressive fibrosing ILD and a short disease duration is attributable to this type of ILD [24].

While comparing the patients that experienced HRQL decline and those who did not, we found that VAS scores were not significantly different between these two groups. We assume that this difference might be explained to some extent by the construction of both questionnaires. VAS asks the patient to rate how they feel on a scale from 1 to 100 today, while K-BILD covers a period of 2 weeks. First, the longer period presumably provides a more realistic picture of average HRQL, which is less affected by episodic highs and lows. We think, thus, VAS is biased upwards to some extent, which is however not the case for K-BILD. Second, the significant K-BILD differences can largely be explained by regression-to-the-mean. Since VAS also contains more general (non-ILD-specific) HRQL restrictions, it is probably only moderately correlated with the K-BILD and is therefore less affected by the regression effect.

The ILD categories (IPF, sarcoidosis and other) differed in terms of disease duration, number of comorbidities and type of medication. To explain these differences, IPF is a deadly disease, characterized by a short disease duration and high number of comorbidities than any other ILD subtype [25]. Regarding medication, apart from patients with IPF-associated cough or acute exacerbations and those who underwent steroid tapering, the majority of IPF patients received anti-fibrotic therapy, while most patients with other ILD subtypes received immunosuppressant medication.

We observed minimal changes in mean K-BILD scores on the population level after a year. These results are similar to results of the INBUILD trial [26], although the trial only recruited patients with progressive fibrosing ILD. Nevertheless, the baseline patient characteristics in this trial were congruent to those in our study, in terms of age, baseline DLCO% predicted and mean K-BILD scores. Baseline K-BILD scores in this study were 52.5 ± 11.0 and 52.3 ± 9.8 in the nintedanib and placebo arms respectively, (52.8 ± 12.1 in our study). After 52 weeks, the measured score differences were under one unit, similar to our study. The authors, however, did not report on the intra-individual changes in HRQL scores according to pre-specified MCIDs, and we could therefore not make a comparison on this level. Nonetheless, we must consider these small changes. Because a third of the patients worsened in HRQL but the mean K-BILD scores remained the same, it is possible that ILD was characterized by alternating good and bad episodes throughout the 12-month period. At the same time, this could be due to a threshold effect. We used the MCID as a threshold for defining deteriorations. Therefore, HRQL decline lower than MCID was defined as stable. As it is the issue with all threshold-based analyses, individuals closely below the threshold resemble those who just passed the threshold, be it from a clinical or a wellbeing-related point of view. To partially mitigate this issue of classifying individuals with similar pattern to different subgroups we performed a sensitivity analysis targeting linear HRQL change, which also captures those individuals which have HRQL decline slightly below MCID.

The present study found a relationship between HRQL decline and lung function decline. This is similar to previous findings. For example, although the study focused on IPF, Kreuter et al. [11] found that an increase in FVC% predicted (ß-coefficient: 0.04, *p* = 0.006) is a predictor for better HRQL scores. Furthermore, a later analysis in the same population also found higher DLCO% predicted to be associated with higher VAS scores [12]. The only study that included different subtypes of ILD did not find any significant impact of lung function with HRQL, but the study was cross-sectional and thus could not detect these long-term associations [27].

Our study revealed alternating HRQL associations with either baseline FVC% predicted or DLCO% predicted in different K-BILD domains, with DLCO% predicted as an important predictor in respiratory domains and FVC% predicted in the psychological domain. Shortness of breath, dyspnea, is generally associated with HRQL decline [10] and with DLCO% predicted in ILD patients [28]. This could explain why DLCO% predicted was associated with ‘breathlessness and activities’ and ‘chest symptoms’ in our study. Similarly, depression is a common comorbidity in ILD [29] and a study found that depressive symptoms correlate with decline of FVC% predicted in ILD patients [30]. We, therefore, demonstrate that lung function parameters predict different aspects of HRQL decline.

Having IPF was linked to less HRQL decline as compared to having sarcoidosis and other ILD subtypes in our study, and there are possible explanations for this. It is known that IPF is associated with substantial morbidity [25] and the prevalence of comorbidities is linked to mortality [22] and HRQL deterioration [11]. In our study, IPF patients had the highest number of comorbidities. We expect that comorbidities, especially in IPF patients, were detected early and managed rapidly, due to the severity of the disease. This in turn could have led to an improvement in overall HRQL. Beyond this, most IPF patients were treated with either nintedanib or pirfenidone. These medications have been shown to slow down lung function decline [6, 31] and we assume IPF patients prescribed either nintedanib or pirfenidone possibly experienced less HRQL deterioration than patients with other ILD subtypes. Another possibility could be survival bias. According to literature, untreated IPF patients have a mean survival time of 3 years [32]. The patients in this cohort had already survived a mean time of 2.4 years and had a survival prognosis of more than another 12 months at the time of recruitment. Therefore, these patients had a better prognosis and HRQL than typical IPF patients. Secondly, patients treated in ILD-expert centers may be assigned an ILD nurse. Studies have shown that ILD nurses have a beneficial role in IPF management [33] and this could have a positive influence on the HRQL of these patients.

We also found higher age to be a determinant of HRQL impairment in ‘psychological impact’ and VAS. It is widely known that functional impairment increases as one ages. This result is consistent with other studies [12].

Although patients with secondary education had a significant association with HRQL decline compared to patients with higher education according to the total K-BILD score, it is possible that the association was due to outliers and the association was random. In this instance, HRQL was not associated with basic education and moreover, the other K-BILD domains were not significantly associated with education, and the direction of the association was not uniform.

Unexpectedly, the use of immunosuppressant medications was not associated with less HRQL decline. However, this association was present with the linear outcome for K-BILD change score in the ‘psychological’ domain. Immunosuppressive agents are used to treat various forms of ILD. It is believed that they improve the lung pathology [4]. We expected patients on immunosuppressive therapy to therefore have better HRQL scores after 12 months.

Another unanticipated finding was comorbidity burden was not a predictor of HRQL decline. Comorbidities may impact treatment decisions and thus impair HRQL [34, 35]. The association between a higher number of comorbidities and HRQL decline was shown in IPF cohorts [12]. Perhaps, comorbidity burden is more significant in IPF than other ILD subtypes. Also, we cannot rule out the effect on HRQL of the medication used to treat comorbidities. On the contrary, however, examining individual comorbidities may have revealed important associations with HRQL decline. Unfortunately, due to the low number of cases, it was not feasible to explore these associations.

We reported a strong association between HRQL decline in K-BILD and study center, which is presumably linked to the center-specific distribution of ILD subtypes in both centers. Patients presenting at the two tertiary care centers for ILD were consecutively recruited; owing to different areas within Germany, the distribution of subtypes differed as described previously [14]. One center included particularly IPF patients, who tend to be older and have a higher comorbidity burden. Despite this center-specific heterogeneity, we decided on a center-adjusted pooled analysis, as stratified analyses resulted in unstable, in part non-converging models for the smaller center. Furthermore, we strongly believe that pooling the data provides a more realistic picture on ILDs in general, as center-specific distributions are averaged to some extent.

Our first sensitivity analysis demonstrated the robustness of our results. Comparable to the main analysis, the main predictors for general HRQL change, DLCO% predicted and baseline HRQL scores predicted similar associations. The second sensitivity analysis excluding IPW showed the complete case analysis underestimated certain predictors of HRQL decline due to drop out bias. The associations that diminished can be explained by the characteristics of the dropouts. They had lower FVC% predicted, and the majority had other ILD subtypes. Moreover, the fact that patients who deteriorated were much older explains the difference of these results.

There are some crucial limitations in our study. This study was observational and therefore, we could only report on associations and not on causations. Furthermore, it is to note that K-BILD does not assess some health issues relevant to ILD such as cough. Cough has been shown to negatively influence HRQL in ILD [36]. However, it is possible that cough was indirectly assessed by its impact on “chest symptoms” and “breathlessness and activities”. In addition, our study did not collect data on weight or body mass index (BMI) and physical activity, and these covariates could provide more insight into the patient’s well-being. A review by Dowman et al. [37] linked improved physical activity in the context of pulmonary rehabilitation with improved HRQL outcomes in ILD patients. Weight loss is common among ILD patients and a study by Pugashetti et al. [38] found an association between weight loss and mortality risk in IPF patients. On the contrary, another study linked higher baseline BMI values to increased frequency of acute exacerbations [39]. It would be interesting to analyze the direction of the effect of weight on HRQL.

Another limitation is the substantial dropout rate of 27.3%, which is however expected in cohort studies on ILDs [40, 41]. This dropout is presumably informative, as the dropouts had lower baseline lung function values than the complete cases which indicates a more severe state of ILD. In consequence, unweighted analyses might underestimate the effect size of lung function. We best possibly addressed the issue of informative dropout by an IPW weighted analysis, and re-affirmed this model by an unweighted analysis (sensitivity analysis 1). Given the high consistency of both approaches, we think that loss-to-follow up might not crucially affect the identified associations.

One strength is that we are the first to apply K-BILD in a non-cross-sectional analysis in a German cohort. We further demonstrate the validity of K-BILD as it captured similar results to the generic VAS. Lastly, although the group is heterogeneous, our study includes otherwise not commonly studied subtypes of ILD, which is important because they have differing prognoses and patterns.

## Conclusion

Around a third of ILD patients experienced a clinically relevant HRQL deterioration in a 12-month period, which was associated with baseline lung function values in all K-BILD domains. Lung function values not only depict disease severity but also effect a person’s well-being, functioning and daily life. As lung function values are time-dependent variables with possible improvements, in contrast to age and ILD subtype, it, thus, seems important to improve lung function and prevent its decline in order to maintain HRQL on the possibly highest level.

## Supplementary information

**Additional file 1: Table S1**. The full spectrum of ILDs in our sample.

**Additional file 2: Table S2**. Characteristics of the complete and incomplete cases.

**Additional file 3: Table S3**. Differences between ILD subtypes regarding disease duration, number of comorbidities and use of immunosuppressant medication.

**Additional file 4: Table S4**. Predictors of HRQL in KBILD total score and VAS with a linear outcome (weighted)

**Additional file 5: Table S5**. Predictors of HRQL deterioration in KBILD total score and VAS (not weighted)

## Data Availability

The data that support the findings of this study are available on reasonable request. The data are not publicly available due to their containing information that could compromise the privacy of research participants.

## Abbreviations

BMI: Body mass index
COPD: Chronic Obstructive Pulmonary Disease
CPFE: Combined pulmonary fibrosis and emphysema
DLCO: Diffusing capacity of carbon monoxide
EQ-5D-5L: EuroQoL 5 dimensions
FVC: Forced vital capacity
HILDA: Health Care in ILD Ambulance Visitors
HP: Hypersensitivity pneumonitis
HRQL: Health-related quality of life
ILD: Interstitial lung diseases
IPF: Idiopathic pulmonary fibrosis
IPW: Inverse probability weighting
K-BILD: King’s Brief Interstitial Lung Disease questionnaire
MCID: Minimal clinically important differences
VAS: Visual Analog Scale
