# Supplementary material for: Determinants of health-related quality of life decline in interstitial lung disease
Source: Health Qual Life Outcomes. 2020 Oct 8;18:334. doi: 10.1186/s12955-020-01570-2 (PMC7542726; doi:10.1186/s12955-020-01570-2)
Supplement: Supplementary file 1 — Additional file 1: Table S1. The full spectrum of ILDs in our sample. [file 12955_2020_1570_MOESM1_ESM.docx]

Additional Table 1: The full spectrum of ILDs in our sample

| ILD Subtype | N (%) |
| --- | --- |
| Idiopathic Interstitial Pneumonias |  |
| - Idiopathic pulmonary fibrosis (IPF) | 52 (26.8) |
| - Non-specific interstitial pneumonia (NSIP) | 10 (5.2) |
| - Desquamative interstitial pneumonia (DIP) | 4 (2.1) |
| - Cryptogenetic organizing pneumonia (COP) | 2 (1.0) |
| - Lymphocytic interstitial pneumonia (LIP) | 1 (0.5) |
| Sarcoidosis | 43 (22.2) |
| Hypersensitivity pneumonitis (exogen allergic alveolitis) | 21 (10.8) |
| Rheumatic and connective tissue diseases with pulmonary involvement | 3 (1.6) |
| Drug-related | 2 (1.0) |
| Combined pulmonary fibrosis and emphysema (CPFE) | 4 (2.1) |
| Other Forms |  |
| - Pulmonary hymphangioleiomyomatosis | 7 (3.6) |
| - Pulmonary Langerhans cell histocytosis | 1 (0.5) |
| - Pulmonary alveolar proteinosis | 2 (1.0) |
| Others | 27 (13.9) |
| Not classifiable | 15 (7.7) |
