# Supplementary material for: Determinants of health-related quality of life decline in interstitial lung disease
Source: Health Qual Life Outcomes. 2020 Oct 8;18:334. doi: 10.1186/s12955-020-01570-2 (PMC7542726; doi:10.1186/s12955-020-01570-2)
Supplement: Supplementary file 2 — Additional file 2: Table S2. Characteristics of the complete and incomplete cases. [file 12955_2020_1570_MOESM2_ESM.docx]

Additional Table 2: Characteristics of the complete and incomplete cases

|  | Complete cases | Incomplete cases | p-value |
| --- | --- | --- | --- |
| N | 194 | 73 |  |
| FVC % predicted, mean (SD) | 74.2 (21.1) | 66.8 (18.4) | **0.009** |
| DLCO% predicted, mean (SD) | 44.5 (16.1) | 42.7 (17.6) | 0.235 |
| K-BILD Breathlessness and Activities, mean (SD) | 41.2 (21.0) | 34.8 (20.4) | 0.082 |
| K-BILD Chest Symptoms, mean (SD) | 62.4 (23.0) | 65.2 (21.9) | 0.339 |
| K-BILD Psychological, mean (SD) | 51.0 (13.9) | 52.5 (13.4) | 0.716 |
| K-BILD Total, mean (SD) | 52.8 (12.1) | 52.5 (10.8) | 0.737 |
| EQ-5D-5L VAS, mean (SD) * | 60.0 (19.5) | 58.8 (19.4) | 0.691 |
| Mean age, years (SD) | 61.7 (12.7) | 63.3 (13.9) | 0.079 |
| Mean time since diagnosis, years (SD) | 4.3 (6.3) | 3.0 (4.8) | **0.026** |
| Mean number of comorbidities (SD) | 2.8 (1.8) | 2.9 (1.6) | 0.442 |
| Female (%) | 66 (34.0) | 25 (34.3) | 0.972 |
| ILD subtypes: |  |  | 0.333 |
| IPF (%) | 55 (28.4) | 15 (20.6) |  |
| Sarcoidosis; % | 43 (22.2) | 15 (20.6) |  |
| Other (%) | 96 (49.5) | 43 (58.9) |  |
| Smoking status: |  |  | 0.923 |
| Current (%) | 7 (3.6) | 2 (2.8) |  |
| Former (%)  Never (%) | 112 (57.7)  75 (38.7) | 43 (59.7)  28 (38.4) |  |
| Immunosuppressant medication use (%) | 117 (60.3) | 49 (67.1) | 0.306 |
| Education: |  |  | 0.888 |
| Basic (%) | 80 (41.2) | 32 (43.8) |  |
| Secondary (%) | 56 (28.9) | 19 (26.0) |  |
| Higher (%) | 58 (29.9) | 22 (30.1) |  |
| Employed (%) | 59 (30.4) | 19 (26.0) | 0.483 |
| Center 2 (%) | 125 (64.4) | 53 (72.6) | 0.207 |

* the complete cases for VAS were 192 and 70 (36.5%) of them had a clinically meaningful decline in HRQL
