# Supplementary material for: Determinants of health-related quality of life decline in interstitial lung disease
Source: Health Qual Life Outcomes. 2020 Oct 8;18:334. doi: 10.1186/s12955-020-01570-2 (PMC7542726; doi:10.1186/s12955-020-01570-2)
Supplement: Supplementary file 3 — Additional file 3: Table S3. Differences between ILD subtypes regarding disease duration, number of comorbidities and use of immunosuppressant medication. [file 12955_2020_1570_MOESM3_ESM.docx]

Additional Table 3: Differences between ILD subtypes regarding disease duration, number of comorbidities and use of immunosuppressant medication

|  | IPF | Sarcoidosis | Other ILDs | p-value |
| --- | --- | --- | --- | --- |
| N | 55 | 43 | 96 |  |
| Mean time since diagnosis, years (SD) | 2.4 (1.8) | 8.1 (9.8) | 3.6 (5.1) | **0.001** |
| Mean number of comorbidities (SD) | 3.6 (1.7) | 1.9 (1.5) | 2.8 (1.7) | **<.0001** |
| Immunosuppressant medications use N (%) | 9 (16.4) | 35 (81.4) | 70 (72.9) | **<.0001** |
