# Supplementary material for: Determinants of health-related quality of life decline in interstitial lung disease
Source: Health Qual Life Outcomes. 2020 Oct 8;18:334. doi: 10.1186/s12955-020-01570-2 (PMC7542726; doi:10.1186/s12955-020-01570-2)
Supplement: Supplementary file 4 — Additional file 4: Table S4. Predictors of HRQL in KBILD total score and VAS with a linear outcome (weighted) [file 12955_2020_1570_MOESM4_ESM.docx]

Additional Table 4: Predictors of HRQL in KBILD total score and VAS with a linear outcome (weighted)

|  | KBILD Total | | | VAS | | |
| --- | --- | --- | --- | --- | --- | --- |
|  | **Beta** | **95 % CI** | **p-value** | **Beta** | **95% CI** | **p-value** |
| FVC % predicted | 0.030 | [-0.031;0.092] | 0.333 | 0.077 | [-0.042;0.197] | 0.205 |
| DLCO % predicted | **0.151** | **[0.061;0.240]** | **0.001** | 0.169 | [-0.003;0.341] | 0.054 |
| Baseline HRQL score | **-0.369** | **[-0.48;-0.258]** | **<0.0001** | **-0.441** | **[-0.575;-0.308]** | **<0.0001** |
| Age | -0.122 | [-0.265;0.021] | 0.095 | -0.090 | [-0.358;0.178] | 0.510 |
| Time since diagnosis | -0.101 | [-0.326;0.125] | 0.382 | -0.046 | [-0.479;0.387] | 0.835 |
| Number of comorbidities | 0.718 | [-0.089;1.525] | 0.081 | -0.405 | [-1.970;1.161] | 0.613 |
| Female | -2.300 | [-4.873;0.274] | 0.080 | -3.358 | [-8.329;1.612] | 0.185 |
| ILD Subtype (ref = Other ILD) |  |  |  |  |  |  |
| IPF | 1.165 | [-2.242;4.572] | 0.503 | 3.586 | [-3.008;10.180] | 0.286 |
| Sarcoidosis | -0.395 | [-4.255;3.466] | 0.841 | -1.679 | [-9.072;5.713] | 0.656 |
| Smoking (ref = non-smoker) |  |  |  |  |  |  |
| Current smoker | 3.971 | [-3.111;11.052] | 0.272 | 2.839 | [-10.746;16.423] | 0.682 |
| Former smoker | 0.746 | [-1.845;3.338] | 0.572 | 0.586 | [-4.448;5.620] | 0.820 |
| Immunosuppressant use | 2.240 | [-0.802;5.281] | 0.149 | 0.500 | [-5.329;6.330] | 0.866 |
| Education (ref = higher) |  |  |  |  |  |  |
| Basic education | 1.374 | [-1.535;4.282] | 0.355 | 1.945 | [-3.815;7.705] | 0.508 |
| Secondary education | -0.633 | [-3.75;2.485] | 0.691 | 3.484 | [-2.536;9.503] | 0.257 |
| Unemployed | -0.407 | [-4.074;3.260] | 0.828 | -6.872 | [-13.831;0.087] | 0.053 |
| Center 2 (ref = center 1) | **-4.366** | **[-7.227;-1.505]** | **0.003** | -1.103 | [-6.612;4.407] | 0.695 |

Additional Table 4 continued: Predictors of HRQL in KBILD domains with a linear outcome (weighted)

|  | KBILD Breathlessness and Activities | | | KBILD Chest | | | KBILD Psychological | | |
| --- | --- | --- | --- | --- | --- | --- | --- | --- | --- |
|  | **Beta** | **95 % CI** | **p-value** | **Beta** | **95 % CI** | **p-value** | **Beta** | **95 % CI** | **p-value** |
| FVC % predicted | -0.022 | [-0.136;0.092] | 0.701 | -0.002 | [-0.127;0.122] | 0.971 | 0.069 | [-0.006;0.144] | 0.071 |
| DLCO % predicted | **0.307** | **[0.143;0.471]** | **<0.0001** | **0.205** | **[0.026;0.384]** | **0.025** | **0.146** | **[0.037;0.255]** | **0.009** |
| Baseline HRQL score | **-0.383** | **[-0.506;-0.260]** | **<0.0001** | **-0.444** | **[-0.556;-0.333]** | **<0.0001** | **-0.446** | **[-0.559;-0.334]** | **<0.0001** |
| Age | -0.092 | [-0.349;0.166] | 0.487 | -0.121 | [-0.406;0.165] | 0.407 | -0.128 | [-0.302;0.046] | 0.149 |
| Time since diagnosis | -0.193 | [-0.607;0.221] | 0.361 | -0.044 | [-0.496;0.408] | 0.850 | -0.136 | [-0.412;0.139] | 0.331 |
| Number of comorbidities | 0.637 | [-0.858;2.133] | 0.404 | 1.284 | [-0.317;2.886] | 0.116 | 0.875 | [-0.096;1.845] | 0.077 |
| Female | -4.194 | [-8.912;0.524] | 0.081 | **-5.618** | **[-10.781;-0.455]** | **0.033** | -1.855 | [-4.996;1.287] | 0.247 |
| ILD Subtype (ref = Other ILD) |  |  |  |  |  |  |  |  |  |
| IPF | 3.979 | [-5.275;13.232] | 0.399 | -1.207 | [-11.299;8.885] | 0.815 | -4.988 | [-11.126;1.15] | 0.111 |
| Sarcoidosis | 2.459 | [-3.796;8.714] | 0.441 | -1.651 | [-8.494;5.193] | 0.636 | -3.057 | [-7.216;1.102] | 0.150 |
| Smoking (ref = non-smoker) |  |  |  |  |  |  |  |  |  |
| Current smoker | 6.725 | [-6.259;19.708] | 0.310 | 6.804 | [-7.423;21.032] | 0.349 | 2.113 | [-6.54;10.767] | 0.632 |
| Former smoker | 1.298 | [-3.434;6.030] | 0.591 | 0.922 | [-4.272;6.116] | 0.728 | 0.812 | [-2.345;3.969] | 0.614 |
| Immunosuppressant use | 0.525 | [-5.053;6.103] | 0.854 | 3.073 | [-3.02;9.166] | 0.323 | **4.463** | **[0.755;8.171]** | **0.018** |
| Education (ref = higher) |  |  |  |  |  |  |  |  |  |
| Basic education | 2.109 | [-3.279;7.497] | 0.443 | -1.566 | [-7.387;4.255] | 0.598 | 1.784 | [-1.759;5.327] | 0.324 |
| Secondary education | -0.747 | [-6.459;4.966] | 0.798 | -4.586 | [-10.836;1.664] | 0.150 | -1.508 | [-5.319;2.304] | 0.438 |
| Unemployed | -1.992 | [-8.684;4.700] | 0.560 | -2.403 | [-9.647;4.841] | 0.516 | -1.481 | [-5.91;2.948] | 0.512 |
| Center 2 (ref = center 1) | **-5.759** | **[-11.007;-0.510]** | **0.032** | **-6.365** | **[-12.101;-0.629]** | **0.030** | **-5.933** | **[-9.423;-2.443]** | **0.001** |
