# Supplementary material for: Determinants of health-related quality of life decline in interstitial lung disease
Source: Health Qual Life Outcomes. 2020 Oct 8;18:334. doi: 10.1186/s12955-020-01570-2 (PMC7542726; doi:10.1186/s12955-020-01570-2)
Supplement: Supplementary file 5 — Additional file 5: Table S5. Predictors of HRQL deterioration in KBILD total score and VAS (not weighted) [file 12955_2020_1570_MOESM5_ESM.docx]

Additional Table 5: Predictors of HRQL deterioration in KBILD total score and VAS (not weighted)

|  | KBILD Total | | | VAS | | |
| --- | --- | --- | --- | --- | --- | --- |
|  | **Beta** | **95% CI** | **p-value** | **Beta** | **95% CI** | **p-value** |
| FVC % predicted | -0.020 | [-0.041;0.002] | 0.070 | **-0.032** | **[-0.053;-0.010]** | **0.004** |
| DLCO % predicted | -0.024 | [-0.054;0.006] | 0.121 | -0.012 | [-0.039;0.015] | 0.384 |
| Baseline HRQL score | **0.099** | **[0.054;0.144]** | **<0.0001** | **0.038** | **[0.017;0.059]** | **<0.0001** |
| Age | 0.015 | [-0.031;0.060] | 0.526 | 0.030 | [-0.011;0.071] | 0.147 |
| Time since diagnosis | -0.008 | [-0.07;0.055] | 0.813 | -0.020 | [-0.083;0.042] | 0.527 |
| Number of comorbidities | 0.041 | [-0.209;0.291] | 0.747 | -0.013 | [-0.246;0.221] | 0.916 |
| Female | 0.713 | [-0.136;1.562] | 0.100 | 0.192 | [-0.568;0.952] | 0.621 |
| ILD Subtype (ref = Other ILD) |  |  |  |  |  |  |
| IPF | -0.989 | [-2.045;0.067] | 0.066 | -0.142 | [-1.076;0.792] | 0.765 |
| Sarcoidosis | 0.012 | [-1.219;1.242] | 0.985 | 0.498 | [-0.653;1.648] | 0.396 |
| Smoking (ref = non-smoker) |  |  |  |  |  |  |
| Current smoker | -0.570 | [-2.738;1.597] | 0.606 | 0.469 | [-1.483;2.421] | 0.638 |
| Former smoker | -0.212 | [-1.009;0.584] | 0.602 | 0.060 | [-0.697;0.817] | 0.877 |
| Immunosuppressant use | -0.421 | [-1.358;0.516] | 0.379 | -0.206 | [-1.059;0.646] | 0.635 |
| Education (ref = higher) |  |  |  |  |  |  |
| Basic education | 0.436 | [-0.529;1.400] | 0.376 | 0.004 | [-0.833;0.841] | 0.993 |
| Secondary education | **1.011** | **[0.017;2.005]** | **0.046** | -0.363 | [-1.270;0.544] | 0.433 |
| Unemployed | 0.014 | [-1.143;1.171] | 0.981 | 0.416 | [-0.653;1.485] | 0.446 |
| Center 2 (ref = center 1) | **1.171** | **[0.218;2.125]** | **0.016** | 0.339 | [-0.479;1.156] | 0.417 |

Additional Table 5 continued**:** Predictors of HRQL deterioration in KBILD domains (not weighted)

|  | KBILD Breathlessness and Activities | | | KBILD Chest | | | KBILD Psychological | | |
| --- | --- | --- | --- | --- | --- | --- | --- | --- | --- |
|  | **Beta** | **95% CI** | **p-value** | **Beta** | **95% CI** | **p-value** | **Beta** | **95% CI** | **p-value** |
| FVC % predicted | 0.005 | [-0.016;0.025] | 0.654 | -0.005 | [-0.026;0.016] | 0.640 | -0.016 | [-0.037;0.006] | 0.147 |
| DLCO % predicted | **-0.034** | **[-0.062;-0.005]** | **0.021** | **-0.037** | **[-0.068;-0.006]** | **0.019** | -0.013 | [-0.044;0.017] | 0.390 |
| Baseline HRQL score | **0.030** | **[0.010;0.050]** | **0.004** | **0.053** | **[0.032;0.074]** | **<.0001** | **0.090** | **[0.050;0.130]** | **<0.0001** |
| Age | 0.010 | [-0.032;0.051] | 0.647 | -0.001 | [-0.049;0.046] | 0.952 | 0.046 | [-0.003;0.095] | 0.065 |
| Time since diagnosis | 0.010 | [-0.051;0.071] | 0.754 | -0.018 | [-0.086;0.050] | 0.602 | 0.027 | [-0.039;0.094] | 0.423 |
| Number of comorbidities | 0.110 | [-0.130;0.349] | 0.369 | 0.020 | [-0.236;0.276] | 0.879 | -0.071 | [-0.323;0.182] | 0.583 |
| Female | 0.643 | [-0.149;1.435] | 0.112 | 0.633 | [-0.234;1.500] | 0.152 | 0.241 | [-0.603;1.085] | 0.576 |
| ILD Subtype (ref = Other ILD) |  |  |  |  |  |  |  |  |  |
| IPF | 0.109 | [-0.858;1.075] | 0.826 | -0.308 | [-1.352;0.736] | 0.563 | **-1.107** | **[-2.163;-0.051]** | **0.040** |
| Sarcoidosis | -0.448 | [-1.614;0.718] | 0.452 | 0.769 | [-0.537;2.076] | 0.248 | -0.243 | [-1.546;1.060] | 0.715 |
| Smoking (ref = non-smoker) |  |  |  |  |  |  |  |  |  |
| Current smoker | -1.285 | [-3.686;1.116] | 0.294 | -0.624 | [-2.872;1.625] | 0.587 | -0.098 | [-2.401;2.205] | 0.934 |
| Former smoker | -0.352 | [-1.092;0.389] | 0.352 | -0.594 | [-1.428;0.240] | 0.163 | -0.185 | [-1.032;0.661] | 0.667 |
| Immunosuppressant use | 0.166 | [-0.715;1.047] | 0.712 | -0.243 | [-1.177;0.691] | 0.610 | -0.478 | [-1.418;0.462] | 0.319 |
| Education (ref = higher) |  |  |  |  |  |  |  |  |  |
| Basic education | -0.177 | [-1.041;0.688] | 0.689 | 0.434 | [-0.545;1.413] | 0.385 | 0.111 | [-0.825;1.047] | 0.816 |
| Secondary education | 0.433 | [-0.448;1.315] | 0.335 | 0.902 | [-0.138;1.943] | 0.089 | 0.357 | [-0.653;1.367] | 0.488 |
| Unemployed | -0.513 | [-1.618;0.592] | 0.363 | 0.833 | [-0.430;2.095] | 0.196 | 0.492 | [-0.754;1.737] | 0.439 |
| Center 2 (ref = center 1) | 0.119 | [-0.732;0.970] | 0.784 | **1.064** | **[0.112;2.016]** | **0.028** | 0.805 | [-0.136;1.747] | 0.093 |
